# Key role of the endothelium in lung ischemia-reperfusion injury: What the clinician needs to know

**DOI:** 10.1016/j.jhlto.2026.100503

**Published:** 2026-01-29

**Authors:** Emmanuel BESNIER, Thomas CLAVIER, Jérémy BELLIEN, Jean SELIM

**Affiliations:** a Department of Anesthesiology and Critical Care, Rouen University Hospital, F-76000, Rouen, France; b Univ Rouen Normandie, INSERM EnVI UMR 1096, F-76000 Rouen, France

**Keywords:** Endothelial dysfunction, Glycocalyx, Ischemia-reperfusion, Oxidative stress, Lung transplantation

## Abstract

Lung transplantation remains the definitive treatment for selected patients with end-stage respiratory failure; however, outcomes are limited by significant early morbidity and mortality. Primary graft dysfunction (PGD), occurring within the first 72 hours after transplantation, is the most severe early complication and is largely driven by pulmonary ischemia–reperfusion (IR) injury. Growing evidence identifies the pulmonary endothelium as a primary target of IR, with endothelial dysfunction playing a central role in increased vascular permeability, pulmonary edema, impaired gas exchange, and altered pulmonary vascular tone.

During ischemia, endothelial metabolic alterations, oxidative stress priming, and glycocalyx degradation sensitize the pulmonary microvasculature to reperfusion injury. Reperfusion then triggers a marked inflammatory response characterized by reactive oxygen species generation, calcium overload, mitochondrial dysfunction, leukocyte recruitment, and disruption of intercellular junctions, leading to endothelial barrier breakdown and microvascular leakage. The endothelial glycocalyx, a critical regulator of vascular permeability and mechanotransduction, is particularly vulnerable to IR injury, and its degradation further amplifies inflammation and capillary leakage.

This narrative review summarizes the key mechanisms involved in pulmonary IR injury, with a focus on endothelial and glycocalyx dysfunction and their contribution to PGD after lung transplantation. We discuss major pathways involved in vasomotor dysregulation, oxidative stress, and inflammatory signaling, as well as the influence of graft preservation strategies, including cold storage temperature and ex vivo lung perfusion. Finally, we review emerging therapeutic approaches aimed at preserving endothelial integrity and glycocalyx structure.

Targeting endothelial and glycocalyx protection represents a promising strategy to reduce IR-related lung injury and improve post-transplant outcomes.

## Introduction

Lung transplantation currently represents the definitive treatment for end-stage respiratory failure in carefully selected patients. It is indicated in cases of end-stage respiratory failure with limited life expectancy and/or poor quality of life in patients under the age of 65. Over the past decades, lung transplantation activity has increased worldwide, with approximately 4000 transplants performed annually compared to 250 in the 1990s.^1^ However, despite the increase in lung transplantation procedures, clinical outcomes remain suboptimal, particularly when compared to other solid organ transplants. Significant challenges persist, with considerable morbidity and mortality. Indeed, although survival varies depending on the underlying disease, the median survival remains 5.3 years.^2^

During the early postoperative period, several complications may threaten the patient, the most significant being primary graft dysfunction (PGD). PGD is characterized by a lung injury-induced edema occurring within the first 72 h after transplantation. PGD is a common complication, with an incidence ranging from 20% to 60% depending on the series. However, despite advancements at every stage of the transplantation process, the incidence of severe PGD has stabilized between 10% and 15% over the past decade.^3^ The etiologies of PGD are multifactorial, with several stages of the transplantation process contributing to its development: (1) brain death, leading to neurogenic pulmonary edema and/or other lung injuries (aspiration pneumonia, low cardiac output); (2) organ retrieval; (3) cold ischemia during transport; (4) ischemia-reperfusion (IR) injury and pulmonary endothelial dysfunction; and (5) recipient's condition.^4^ These stages are pathophysiologically interconnected in PGD, making it difficult to identify a single predominant cause. However, they represent key targets for potential therapeutic interventions aimed at reducing its incidence.^5^ In particular, increasing evidence shows that PGD primarily results from IR injury and the associated pulmonary endothelial dysfunction, making the understanding of the underlying pathophysiological mechanisms a major research focus, with the ultimate goal of reducing post-transplant morbidity and mortality.^6^

Pulmonary endothelium is the primary target of IR, leading to major impairment in its regulatory functions. This alteration disrupts the alveolo-capillary barrier, promoting pulmonary edema, impaired gas exchange, and reduced pulmonary compliance.^7^ IR also triggers an inflammatory cascade, involving the recruitment of immune cells and the release of damage-associated molecular patterns (DAMPs) and reactive oxygen species (ROS) by various cell types, including pulmonary endothelial cells. This inflammation facilitates leukocyte migration and adhesion, which in turn leads to the secretion of inflammatory mediators responsible for further endothelial injury. Numerous studies have demonstrated that IR injury and pulmonary endothelial dysfunction play a key role in graft dysfunction**.** Consequently, several therapeutic strategies have been explored to mitigate these detrimental effects.^8^

The objective of this narrative review is to summarize the main mechanisms involved in pulmonary IR injury, with a focus on the key roles of the endothelium and its covering layer named glycocalyx—particularly in the regulation of vasomotor tone—and to discuss their contribution to lung dysfunction after pulmonary transplantation. Finally, we will discuss several therapeutic agents that have been investigated for their potential to preserve endothelial function and protect the glycocalyx.

## The endothelium

Previously regarded as an inert and passive structure, the vascular endothelium is now recognized as a fully functional, highly specialized, and metabolically active organ. It plays a key role in numerous physiological, immunological, and synthetic processes, including vasomotor tone regulation, intravascular coagulation, leukocyte extravasation, inflammation, vascular permeability, and the production of chemokines, cytokines, growth factors, and ROS. In addition to its barrier and metabolic functions, it also contributes to the expression of surface receptors, signaling molecules, and adhesion proteins involved in cellular communication and immune responses.^9^, ^10^ The endothelium lines the innermost layer of blood vessels and consists of a monolayer of mesenchyme-derived endothelial cells, a subcellular extracellular matrix, and a luminal glycocalyx. The glycocalyx acts as a semi-permeable barrier separating blood from the underlying tissue—particularly lung tissue—and regulates the trafficking of proteins, leukocytes, and fluids.^11^ Furthermore, components of the endothelial cell membrane can sense changes in shear stress and hemodynamic pressure. In the pulmonary endothelium, mechanical forces generated by blood flow and lung inflation are translated into intracellular signals through mechanotransduction mechanisms (including shear stress–activated ion channels, integrin–cytoskeleton coupling, and membrane-associated mechanosensors). These signals regulate calcium-dependent pathways (e.g., Ca²⁺ influx and downstream kinase activation), cytoskeletal remodeling (actin reorganization and cell stiffness), and redox-sensitive signaling (NO/ROS balance), thereby influencing endothelial metabolism, gene expression, and structural integrity. Through these adaptive mechanisms, mechanical stimuli contribute to pulmonary vascular homeostasis and shape endothelial responses under pathological conditions.^12^

Endothelial cells form the boundary between the blood and the vascular wall. They have a rhomboid shape and are arranged in a mosaic-like pattern. They rest on a basement membrane primarily composed of glycoproteins and collagen. Various types of junctions exist between endothelial cells, allowing them to juxtapose, interlock, or overlap. These junctional differences enable the endothelium to regulate and adapt to the passage of plasma proteins. Tight junctions provide a selective barrier, whereas gap junctions facilitate communication between adjacent cells, allowing the exchange of regulatory factors, metabolites, and ions. Intercellular junctions—specifically tight junctions and adherens junctions—between adjacent endothelial cells are essential for maintaining endothelial integrity.^13^ These junctions ensure vascular barrier function and modulate signal transduction through dynamic interactions with cytoskeletal structures, including actin microfilaments and microtubules, particularly in response to shear stress and mechanical forces applied to the endothelium. Additionally, membrane-bound integrins anchor the endothelial monolayer to focal adhesion plaques, reinforcing the structural stability of the vascular lining. Notably, increased vascular permeability and the development of edema are primarily associated with dysfunction of tight junctions.^14^ Furthermore, interendothelial junctions are covered by the endothelial glycocalyx, a protective luminal layer whose disruption also contributes significantly to capillary leakage and barrier breakdown.^15^ Given its multifaceted roles in metabolism, mechanosensing, and vascular homeostasis, the endothelium is particularly vulnerable to IR injury.^16^ Ischemia triggers a local pro-inflammatory response that sensitizes the endothelium to further damage upon reperfusion. During ischemia, endothelial metabolism shifts toward purine degradation, leading to the accumulation of hypoxanthine and xanthine, while enzymatic systems such as xanthine oxidoreductase become primed for reactive oxygen species generation. This ischemic milieu promotes early oxidative stress, glycocalyx degradation, and endothelial activation, favoring the local expression and binding of pro-inflammatory mediators and adhesion molecules. As a result, the endothelium becomes sensitized to reperfusion, amplifying inflammatory signaling, leukocyte recruitment, and microvascular dysfunction. The reperfusion phase then induces a robust inflammatory cascade, both locally and systemically, leading to the disassembly of intercellular junctions, formation of paracellular gaps, and increased transcellular permeability through the altered expression of transmembrane ion channels. In particular, alterations in calcium (Ca²⁺) influx pathways, potassium (K⁺) channel conductance, and sodium (Na⁺) transport disrupt endothelial cytoskeletal organization and junctional stability, thereby exacerbating barrier dysfunction and microvascular leakage. Oxidative stress, ROS—produced by activated endothelial cells and infiltrating leukocytes—are major contributors to endothelial degradation and dysfunction.^17^

A key characteristic of the endothelium is its remarkable plasticity. Endothelial cells can dynamically modulate their structure and function in response to chemical or physical stimuli, such as blood flow. Mechanotransduction refers to the process by which mechanical forces sensed by mechanoreceptors are converted into intracellular biochemical signals. These signals regulate key cellular processes such as gene expression, migration, proliferation, and differentiation. In this context, the endothelial surface glycocalyx acts as a critical mechanosensor that detects shear stress and transmits these mechanical cues to the underlying endothelial cells. This mechanosensing activates membrane-associated signaling involving ion channel modulation (K^+^ channel closure and Ca²⁺ influx), membrane depolarization, and downstream kinase pathways such as PI3K/Akt, ultimately driving redox signaling and endothelial functional adaptation.^11^

### Pulmonary endothelium role

Due to their strategic location at the interface between the bloodstream and lung tissue, pulmonary endothelial cells play a key role in optimizing gas exchange, maintaining barrier integrity and function, and regulating pulmonary vascular tone. This regulation is mediated through the nitric oxide (NO), prostacyclin (PGI2), endothelin (ET), and serotonin (5-HT) pathways.^18^ Similar to the endothelium in other organs, the pulmonary endothelium is a highly dynamic interface that senses and integrates chemical, physical, and mechanical cues, leading to the release of specific mediators that regulate vasomotor tone and preserve the homeostatic balance between the vascular and parenchymal compartments.^19^ Pulmonary endothelial cells synthesize various paracrine and endocrine factors, enabling them to regulate vascular tone by either promoting relaxation (via PGI2 and NO) or contraction (via ET-1 and 5-HT) under physiological conditions or in response to stimuli. Studies confirm a continuous release of NO, which contributes to the normally low pulmonary vascular tone in normoxia.^20^ During acute hypoxia, eNOs (endothelial nitric oxide synthase) activity increases to modulate and adapt the pulmonary vasomotor response. NO deficiency may also enhance mitogenesis and vascular wall cell proliferation.^21^ Additionally, the RhoA/Rho-associated kinase (ROCK) pathway plays a crucial role in pulmonary vascular tone regulation, through NO-dependent and NO-independent mechanisms.^22^ Hypoxia-induced ROCK activation enhances Ca^2+^ sensitivity of pulmonary endothelial smooth muscle cell myofilaments, increasing vascular tone and, ultimately, pulmonary vascular resistance.

### Regulation of vasomotor tone

**Figure 1:**
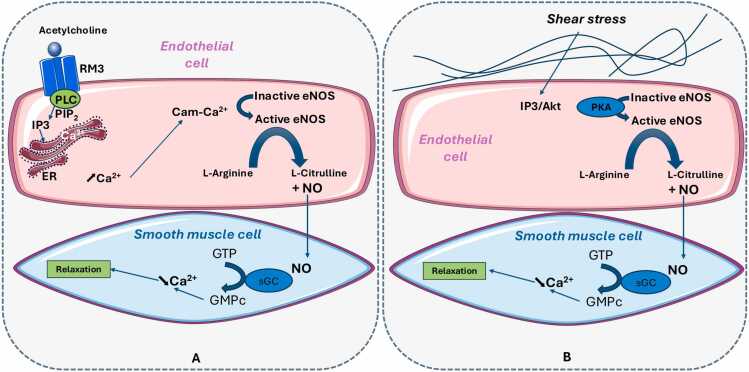
A Calcium-dependent pathway. Acetylcholine binds to its endothelial muscarinic receptor (RM3), leading to the activation of phospholipase C (PLC). PLC activation results in the hydrolysis of Phosphatidylinositol 4,5-bisphosphate, (PIP_2_)), thereby triggering the production of inositol trisphosphate (IP3). IP3 then mobilizes intracellular calcium stores from the endoplasmic reticulum (ER), leading to an increase in intracellular calcium concentration. This rise in calcium, through its binding to calmodulin (Cam), activates endothelial nitric oxide synthase (eNOS), resulting in NO production. The newly synthesized NO diffuses into the underlying layers of the endothelium and activates soluble guanylate cyclase (sGC) in the smooth muscle cells of the media. This activation catalyzes the conversion of GTP into cyclic GMP (cGMP), which subsequently activates protein kinase G (PKG). PKG reduces cytosolic free calcium levels and inhibits smooth muscle cell contraction by promoting myosin dephosphorylation, ultimately leading to vasodilation. B Calcium-independent pathway. eNOS activation is primarily induced by shear stress or blood flow. This process involves protein kinase–dependent pathways, notably the PI3K/Akt and protein kinase A (PKA) pathways, which phosphorylate eNOS and thereby regulate its activity. eNOS activation subsequently leads to NO production, inducing vascular smooth muscle relaxation.

The vasomotor tone is primarily regulated by the endothelium in response to physical or chemical stimuli from the bloodstream or surrounding tissues. The endothelium can release vasodilatory mediators such as NO and prostacyclin (PGI2), as well as vasoconstrictive mediators like platelet-activating factor (PAF) and endothelin. The main endothelial-derived vasorelaxant is NO.^23^ NO is produced through the oxidation of L-arginine to L-citrulline by eNOS, which is calcium-dependent and regulated by phosphorylation.^24^ The reaction follows this pathway: L-arginine + O₂ → L-citrulline + NO. eNOS can be stimulated via specific receptors by bradykinin, substance P, adenosine diphosphate (ADP), muscarinic agonists, or shear stress. For instance, during physical exercise, increased cardiac output leads to enhanced shear stress, which in turn activates eNOS and promotes vasodilation.^25^ eNOS is predominantly expressed in endothelial cells, and its activity is modulated through complex biochemical processes involving multiple regulatory proteins. The common pathway underlying this phenomenon is the production of NO, which diffuses into the deeper layers of the vessels and activates soluble guanylate cyclase in the smooth muscle cells of the media. This activation leads to the formation of cyclic guanosine monophosphate (cGMP), which subsequently activates protein kinase G. This kinase reduces cytosolic free calcium levels and inhibits smooth muscle cell contraction by dephosphorylating myosin.^26^ Two distinct pathways regulate this process: the calcium-dependent pathway and the calcium-independent pathway. The calcium-dependent pathway involves the following mechanisms: under basal conditions, endothelial eNOS is anchored to the plasma membrane within endothelial cell caveolae. When a ligand, such as acetylcholine, binds to its receptor, phospholipase C is activated, triggering the production of inositol trisphosphate (IP3), which mobilizes intracellular calcium stores. The increase in intracellular calcium concentration (via calcium-calmodulin binding) subsequently activates eNOS, leading to NO production 27, 28 **(**Figure 1A**)**. Calcium-independent eNOS activation is primarily involved in NO production in response to shear stress or blood flow. Protein kinases play a key role in this process, particularly the PI3K/Akt (phosphoinositide 3-kinase/protein kinase B) and protein kinase A (PKA) pathways, which phosphorylate eNOS, leading to its activation **(**Figure 1B**)**. It is crucial to understand that during ischemia, the reduction in blood flow leads to a decreased production of NO by the endothelium, primarily through eNOS. Upon reperfusion, there is an excessive production of ROS, particularly superoxide radicals, which react with NO to form peroxynitrite (ONOO-), a pro-inflammatory and cytotoxic compound. This process reduces NO bioavailability and exacerbates endothelial dysfunction. Consequently, vascular tone regulation is also impaired. The clinical consequences include persistent hypoxia despite reperfusion, paradoxical pulmonary vasoconstriction, and pulmonary edema due to increased vascular permeability.

## The glycocalyx

### The role of the pulmonary glycocalyx in ischemia-reperfusion

The glycocalyx forms the luminal surface of the endothelium and is covered by a thin layer of proteoglycans and glycosaminoglycans**.** It has become a major focus of research, particularly in the pulmonary system, where it serves a protective role that may be crucial during IR injury.^29^ As a key vascular barrier, the preservation of the glycocalyx is an important therapeutic target in lung transplantation. The degradation products of the glycocalyx, such as syndecan-1 and heparan sulfate, can be measured and may serve as biomarkers of endothelial injury during IR.^30^

### Structure of the glycocalyx

**Figure 2:**
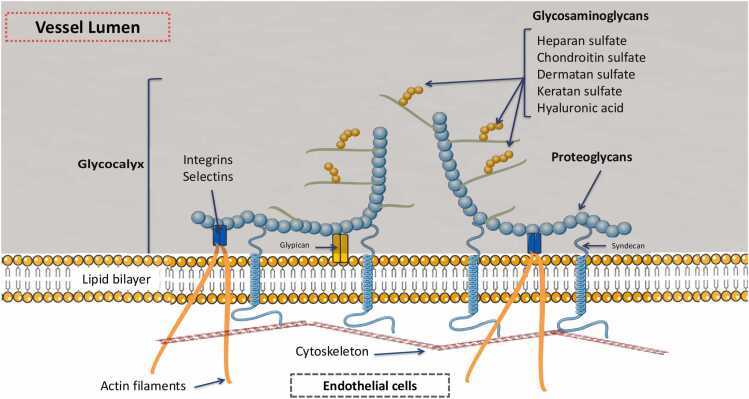
Structural organization of the glycocalyx.

The glycocalyx is a membrane primarily composed of carbohydrates, closely associated with the endothelium. Its various components are interconnected through proteoglycans and glycosaminoglycans. It is a highly dynamic structure, continuously evolving through a balance between synthesis and degradation. The composition of the glycocalyx undergoes constant modulation in response to environmental factors and pathophysiological conditions, such as shear stress, circulatory assistance, sepsis, and IR.^31^ Its degradation is mainly driven by enzymatic reactions and shear stress (Figure 2).

• Proteoglycans. Proteoglycans form the structural backbone of the glycocalyx and can be considered its "spinal column". They consist of core proteins covalently linked to glycosaminoglycans (GAGs). The protein component is anchored to the plasma membrane either via a transmembrane domain (syndecans) or through a lipid anchor (glypicans).
• Glycosaminoglycans. There are five major glycosaminoglycans (GAG), which are variable-length disaccharide polymers: heparan sulfate, dermatan sulfate, chondroitin sulfate, hyaluronic acid (hyaluronan), and keratan sulfate.^32^ These GAGs can undergo acetylation or sulfation modifications. Among them, chondroitin sulfate and heparan sulfate are the most abundantly expressed in endothelial cells.
• Glycoproteins. Glycoproteins also play a fundamental structural role in the architecture of the glycocalyx, as they connect it to the endothelial cell membrane. The three major families of glycoproteins present in the glycocalyx are selectins, integrins, and immunoglobulins.^33^ Selectins (E-selectin and P-selectin) are primarily involved in leukocyte-endothelial cell interactions. It is now well established that pro-inflammatory mediators, such as tumor necrosis factor (TNF-α) and interleukin-1β (IL-1β), increase the expression of selectins on the endothelial surfaced.^34^ The best-characterized immunoglobulins in the glycocalyx include intercellular adhesion molecules-1 and −2 (ICAM-1 and −2), vascular cell adhesion molecule-1 (VCAM-1), and platelet-endothelial cell adhesion molecule-1 (PECAM-1). These immunoglobulins act as ligands for leukocyte and platelet integrins, facilitating their anchoring to the endothelium. This interaction plays a crucial role in the rolling, adhesion, and transendothelial migration (diapedesis) of leukocytes. Additionally, the glycocalyx contains immunoglobulins involved in coagulation, fibrinolysis, and hemostasis.^35^

### Functions of the glycocalyx

Role in Vascular Permeability. The glycocalyx plays a key role in vascular permeability, acting as a regulator that modulates the access of specific molecules to the vascular membrane.^36^ It is well established that the loss of this function leads to interstitial edema, even if the endothelium remains structurally intact. Recent insights into the glycocalyx’s role have led to revisions of Starling’s law, which traditionally described capillary exchange based on the balance between hydrostatic and oncotic pressures across the capillary membrane. The revised model now incorporates the glycocalyx layer as a crucial determinant of transvascular fluid dynamics.

Role in Mechanotransduction. Blood flow exerts mechanical forces on the endothelial lining of blood vessels. These shear stress forces on exposed endothelial cells stimulate the production of NO, a key molecule responsible for vascular reactivity, as discussed in the previous chapter. The mechanisms linking biomechanical signals (shear stress) to biochemical responses (NO synthesis) remain incompletely understood. However, several studies suggest that the glycocalyx may serve as a key mediator in these processes.^37^, ^38^

## Cold ischemia and hypoxia

**Figure 3:**
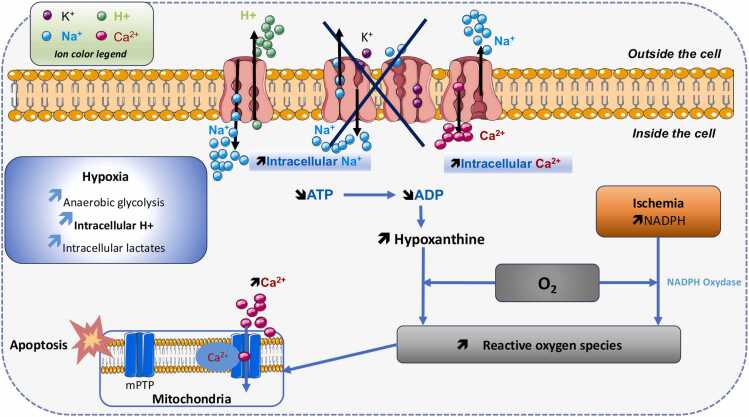
Formation of reactive oxygen species during pulmonary ischemia-reperfusion. Hypoxia at the cellular level induces anaerobic glycolysis, leading to the accumulation of H^+^ ions and hyperlactatemia. A reduction in adenosine triphosphate (ATP) production, coupled with the accumulation of its degradation products, contributes to cellular energy failure. Accumulated H^+^ ions are exchanged with extracellular Na+ ions through the Na^+^/H^+^ exchanger. Due to ATP depletion, the Na^+^/K^+^ pump becomes non-functional, leading to intracellular Na^+^ accumulation and a subsequent increase in intracellular Ca^2+^ via the Na^+^/Ca^2+^ exchanger. The rise in intracellular Ca^2+^ triggers the opening of mitochondrial permeability transition pores (mPTP). Additionally, ischemia leads to the accumulation of Hypoxanthine and nicotinamide adenine dinucleotide phosphate (NADPH), which, through the activation of NADPH oxidase, drives the production of reactive oxygen species (ROS). These ROS further contribute to mPTP opening, exacerbating mitochondrial dysfunction and ultimately triggering apoptosis in pulmonary cells.
• Inactivation of the Sodium Pump. During ischemia, the increase in anaerobic glycolysis leads to an accumulation of H^+^ ions and cytoplasmic hyperlactatemia. The Na^+^/H^+^ exchanger facilitates the removal of H^+^ ions, while sodium is expelled from the cell via the Na^+^/H^+^ ATPase pump. However, under hypothermic or hypoxic conditions, Na^+^/K^+^ ATPase function is impaired, resulting in intracellular sodium accumulation and subsequent cellular edema^39^ **(**Figure 3**)**.
• Intracellular Calcium Overload. Hypoxia leads to the inactivation of the Na^+^/K^+^ exchanger, promoting cytoplasmic calcium accumulation, while hypothermia induces the release of intracellular Ca^2+^. This calcium overload facilitates the opening of mitochondrial permeability transition pores (mPTP) and leads to cellular apoptosis **(**Figure 3**)**.
• Nitric Oxide Production. Nitric oxide synthase exists as three distinct isoforms with specific cellular localizations and functions. The neuronal isoform (nNOS) is mainly found in the cytoplasmic compartment of central nervous system neurons, where it contributes to neuromodulation. The inducible isoform (iNOS) is expressed in immune cells such as macrophages following stimulation by pro-inflammatory cytokines or endotoxins and is primarily associated with cytotoxic responses. The endothelial isoform (eNOS) is largely associated with the endothelial cell membrane and mediates nitric oxide–dependent vascular relaxation. All three isoforms produce NO and require calcium for their activation. During hypoxia, the activity of eNOS, which relies on calcium/calmodulin, is reduced, leading to decreased NO production. This results in vasoconstriction, which is detrimental to the pulmonary parenchyma.^41^ All these steps ultimately lead to pulmonary cell apoptosis and the release of pro-inflammatory mediator.^42^

When a lung is selected for transplantation, it is placed in a cold preservation solution and stored under hypothermic conditions to slow down its metabolism. This phase, known as cold ischemia, corresponds to the organ's cooling period. Ischemia reduces the oxygen supply to the organ via the perfusate, leading to hypoxia. Hypothermia, ischemia, hypoxia, and subsequent reperfusion activate multiple pathophysiological pathways that ultimately result in cell death. These pathways primarily involve (1) oxidative stress, (2) sodium pump inactivation, (3) increased intracellular calcium levels, and (4) NO production^39^:

• Oxidative Stress. Cold ischemia and hypoxia lead to the production of oxygen-derived species such as hydrogen peroxide, superoxide anion, and hydroxyl radicals, which contribute to oxidative stress. The damage induced by oxidative stress ranges from increased permeability to cell lysis and affects the entire pulmonary parenchyma. All cells within the pulmonary parenchyma are impacted by oxidative stress^40^ **(**Figure 3**)**.

## Consequences of reperfusion

### Increased oxidative stress

Hypoxanthine and nicotinamide adenine dinucleotide phosphate (NADPH), which accumulate during cold ischemia and hypoxemia, promote the generation of ROS upon reperfusion and reoxygenation. ROS induce direct cellular injury by oxidizing membrane lipids, structural and enzymatic proteins, and nucleic acids, thereby impairing cellular integrity and function. In parallel, oxidative damage to mitochondrial components promotes mPTP opening, amplifying mitochondrial dysfunction and cell death signaling pathways.^43^

### Cell death and mitochondrial permeability transition pores

Following successful lung transplantation in humans, it is estimated that 25% of pulmonary cells undergo apoptosis within the first two hours after reperfusion. Apoptosis does not occur during ischemia but is triggered upon reperfusion. Cellular apoptosis follows two main pathways: the intrinsic and extrinsic pathways. The intrinsic pathway involves the activation of mPTP and is triggered by intracellular calcium overload and ROS production. The extrinsic pathway is mediated by death receptors and their ligands, including TNF-α and its receptor. The intrinsic pathway is activated early during reperfusion, whereas the extrinsic pathway may take several hours to initiate^44^ **(**Figure 1).

**Figure 4:**
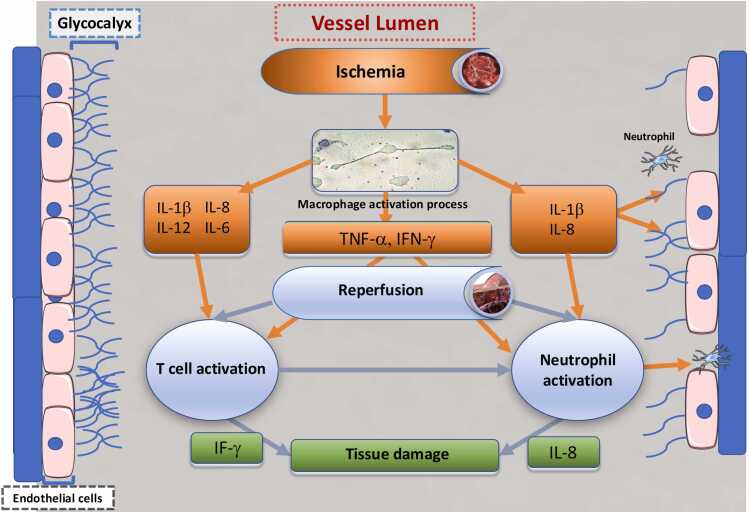
Inflammatory mediators release during pulmonary ischemia-reperfusion.

Release of inflammatory mediators during **IR**. During the acute phase of pulmonary ischemia, as observed in other organs such as the kidney, heart, and liver, alveolar macrophages rapidly release pro-inflammatory cytokines in response to oxidative stress. These include interleukins (IL-1β, IL-6, IL-8, IL-12), interferon-γ (IFN-γ), and TNF-α. These pro-inflammatory cytokines subsequently activate T lymphocytes and neutrophils, which in turn secrete additional IL-8 and IFN-γ, perpetuating the inflammatory cascade^45^, ^46^ **(**Figure 4**)**.

## Therapeutics ways

Significant efforts in both clinical and preclinical research have focused on elucidating the mechanisms leading to endothelial and glycocalyx degradation following pulmonary IR, with the aim of identifying potential therapeutic targets. Simple, non-pharmacological interventions—such as controlled reperfusion and controlled ventilation strategies, particularly the avoidance of hypercapnia—have shown promise in limiting IR-induced injury.^39^, ^47^ In porcine models, sevoflurane preconditioning has been shown to preserve the pulmonary endothelial glycocalyx by limiting the shedding of key structural components such as heparan sulfate and syndecan, likely through attenuation of lysosomal protease activity and inflammatory signaling. In contrast, propofol anesthesia is associated with increased glycocalyx degradation and enhanced expression of chemokines and adhesion molecules following reperfusion.^30^, ^48^ Calcium channel blockers have also been evaluated, showing beneficial effects on inflammation—reflected by decreased IL-6 and TNF-α levels—as well as on vascular permeability.^49^ Additionally, tyrosine kinase inhibitors, initially developed for leukemia treatment, have been associated with reduced vascular leakage.^50^, ^51^ Conversely, rodent models have indicated that circulatory support during IR may exacerbate endothelial dysfunction and glycocalyx damage; however, this effect appears attenuated with the administration of albumin.^27^, ^28^ Finally, ex vivo lung perfusion (EVLP), originally developed for graft reconditioning, is increasingly recognized as a potential therapeutic platform.^52^, ^53^ Multiple studies have demonstrated its ability to serve as a vector for targeted drug delivery aimed at mitigating IR-related injury.^54^, ^55^

## Endothelial and glycocalyx responses to preservation temperature

### 4 °C cold storage versus 10 °C controlled hypothermic storage

Conventional static cold storage at 4 °C, particularly when ice-based, exposes the pulmonary endothelium to near-freezing temperatures that induce cold-related cellular stress rather than selective metabolic suppression. Experimental and translational studies demonstrate that such conditions promote membrane instability, ionic imbalance, mitochondrial dysfunction, and enhanced oxidative stress upon reperfusion. These processes are potent drivers of endothelial activation, junctional disassembly, and activation of proteolytic and oxidative pathways known to mediate glycocalyx shedding, including the loss of heparan sulfate and syndecan components. In contrast, controlled hypothermic storage at 10 °C preserves a reduced yet active aerobic metabolism, maintains mitochondrial integrity, and limits oxidative and inflammatory signaling. By attenuating endothelial activation and reducing triggers of glycocalyx degradation, preservation at 10 °C is therefore expected to better maintain endothelial barrier function and glycocalyx integrity compared with conventional 4 °C storage.^56^, ^57^, ^58^, ^59^

### 10 °C controlled hypothermic storage versus EVLP

Compared with EVLP-based reconditioning strategies, controlled hypothermic storage at 10 °C avoids exposing the endothelium to normothermic perfusion prior to implantation. While EVLP provides functional assessment and optimization, normothermic perfusion is associated with endothelial activation, shear stress–dependent mechanotransduction, oxidative stress, and inflammatory mediator release, all of which have been implicated in glycocalyx shedding and endothelial surface layer disruption. Moreover, repeated temperature transitions inherent to EVLP protocols (cold–warm–cold) may further sensitize the endothelial glycocalyx to subsequent ischemia–reperfusion injury. In contrast, continuous preservation at 10 °C could minimizes thermal fluctuations and inflammatory priming, thereby potentially limiting endothelial activation and preserving glycocalyx integrity prior to implantation.^60^, ^61^

## Conclusion

Pulmonary IR is a complex process that triggers a massive inflammatory response, leading to lung dysfunction, oxidative stress, endothelial barrier disruption, increased vascular permeability, alveolar damage, and leukocyte infiltration. The loss of endothelial barrier function and the resulting edema are driven by two distinct events occurring during IR: pulmonary hyperpermeability and neutrophil infiltration. Numerous mediators of endothelial dysfunction have now been identified and have become targets for therapeutic interventions. The literature provides strong evidence supporting the critical role of the endothelium and glycocalyx in IR injury, highlighting their potential as key therapeutic targets.

## Ethics Committee number

Not applicable.

## Funding Statement

Support was provided solely from departmental sources.

## Clinical trial registration number

Not applicable.

## Data Availability Statement

Bot applicable.

## Declaration of Competing Interest

The authors declare that they have no known competing financial interests or personal relationships that could have appeared to influence the work reported in this paper.
